# Spexin role in human granulosa cells physiology and PCOS: expression and negative impact on steroidogenesis and proliferation^†^

**DOI:** 10.1093/biolre/ioad108

**Published:** 2023-09-02

**Authors:** Patrycja Kurowska, Monika Dawid, Julia Oprocha, Natalia Respekta, Loïse Serra, Anthony Estienne, Piotr Pawlicki, Małgorzata Kotula-Balak, Fabrice Guérif, Joelle Dupont, Agnieszka Rak

**Affiliations:** Laboratory of Physiology and Toxicology of Reproduction, Institute of Zoology and Biomedical Research, Jagiellonian University in Krakow, Krakow, Poland; Laboratory of Physiology and Toxicology of Reproduction, Institute of Zoology and Biomedical Research, Jagiellonian University in Krakow, Krakow, Poland; Doctoral School of Exact and Natural Sciences, Jagiellonian University in Krakow, Krakow, Poland; Laboratory of Physiology and Toxicology of Reproduction, Institute of Zoology and Biomedical Research, Jagiellonian University in Krakow, Krakow, Poland; Laboratory of Physiology and Toxicology of Reproduction, Institute of Zoology and Biomedical Research, Jagiellonian University in Krakow, Krakow, Poland; Doctoral School of Exact and Natural Sciences, Jagiellonian University in Krakow, Krakow, Poland; National Research Institute for Agriculture, Food and the Environment, UMR85, Unité Physiologie de la Reproduction et des Comportements, Nouzilly, France; National Research Institute for Agriculture, Food and the Environment, UMR85, Unité Physiologie de la Reproduction et des Comportements, Nouzilly, France; Center of Experimental and Innovative Medicine, University of Agriculture in Krakow, Krakow, Poland; Department of Animal Anatomy and Preclinical Sciences, University Centre of Veterinary Medicine JU-UA, University of Agriculture in Krakow, Krakow, Poland; National Research Institute for Agriculture, Food and the Environment, UMR85, Unité Physiologie de la Reproduction et des Comportements, Nouzilly, France; Reproductive Medicine and Biology Department, University Hospital of Tours, Tours, France; National Research Institute for Agriculture, Food and the Environment, UMR85, Unité Physiologie de la Reproduction et des Comportements, Nouzilly, France; Laboratory of Physiology and Toxicology of Reproduction, Institute of Zoology and Biomedical Research, Jagiellonian University in Krakow, Krakow, Poland

**Keywords:** spexin, granulosa, proliferation, steroidogenesis, signaling pathways, galanin receptors, polycystic ovarian syndrome

## Abstract

Spexin (SPX) is a novel neuropeptide and adipokine negatively correlated with obesity and insulin resistance. A recent study investigated expression and regulatory function of SPX in the hypothalamus and pituitary; however, the effect on ovarian function is still unknown. The aim of this study was to characterize the expression of SPX and its receptors, galanin receptors 2 and 3 (GALR2/3), in the human ovary and to study its in vitro effect on granulosa cells (GC) function. Follicular fluid (FF) and GC were obtained from normal weight and obese healthy and diagnosed with polycystic ovarian syndrome (PCOS) women. Expression of SPX and GALR2/3 in the ovary was studied by qPCR, western blot, and immunohistochemistry. The level of SPX in FF was assessed by enzyme-linked immunosorbent assay. The in vitro effect of recombinant human SPX on GC proliferation, steroidogenesis, and signaling pathways (MAP3/1, STAT3, AKT, PKA) was analyzed. Moreover, GC proliferation and estradiol (E2) secretion were measured with and without an siRNA against GALR2/3 and pharmacological inhibition of the above kinases. The results showed that both the SPX concentration in FF and its gene expression were decreased in GC of obese and PCOS women, while the protein expression of GALR2/3 was increased. We noted that SPX reduced GC proliferation and steroidogenesis; these effects were mediated by GALR2/3 and kinases MAP3/1, AKT, and STAT3 for proliferation or kinases MAP3/1 and PKA for E2 secretion. The obtained data clearly documented that SPX is a novel regulator of human ovarian physiology and possibly plays a role in PCOS pathogenesis.

## Teaser

We observed decreased spexin (SPX) levels in granulosa cells (GC) and follicular fluid collected from obese and polycystic ovarian syndrome women, and we noted an inhibitory effect of SPX on GC proliferation and steroidogenesis.

## Graphical Abstract

**Graphical Abstract:**
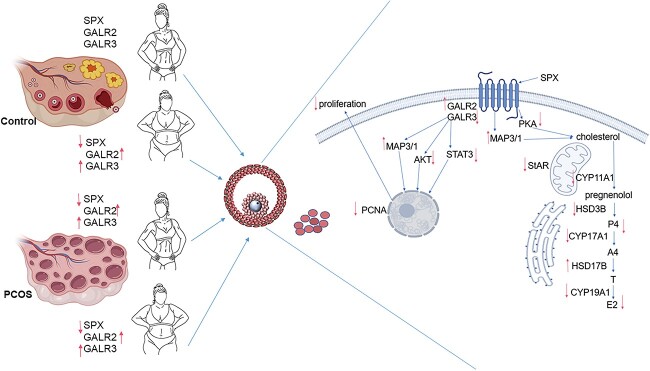

## Introduction

Spexin (SPX), a novel neuropeptide and adipokine, was discovered in 2007 using a bioinformatics method based on a hidden Markov model [1]. In humans, its protein is encoded by the RNA 5'-phosphate and 3'-OH ligase 1 (*RLIG1*, previously known as *C12ORF29*) gene, which is located on chromosome 12 and consists of six exons and five introns. This gene encodes a SPX prepropeptide of 116 amino acids, while an active neuropeptide is built from a 14-amino-acid polypeptide [1]. Expression of SPX was described mainly in human white adipose tissue [1], but it was also found in the brain, heart, lung, liver, pancreas, stomach, and diverse sections of the gastrointestinal tract, as reported in both fish and mammals [2, 3]. Structural analysis of SPX showed that it belongs to the Spexin/Galanin/Kisspeptin gene family [4]. Moreover, it was demonstrated that SPX activates galanin (GAL) receptors type 2 (GALR2) and 3 (GALR3) [4]. It has been demonstrated that SPX may modulate many processes by GALR activation, including stimulating intestinal/colonic smooth muscle contractions via GALR2 in mice [5] or reducing hepatic and circulating levels of total bile acids via GALR2 and GALR3 in rats [6]. Interestingly, the effect of SPX on the regulation of energy homeostasis, appetite control, and fatty acid uptake has already been described [7]. Levels of SPX in adipose tissue were remarkably decreased in obese individuals [8, 9], and injection of SPX into the brain inhibited food consumption and decreased the expression of orexigenic factors in goldfish [10]. Moreover, SPX significantly impeded fatty acid uptake into the adipocytes in mice and humans [9], as well as down-regulating the expression of pro-adipogenic genes while increasing lipolysis in mice [11]. Furthermore, SPX treatment improved glucose tolerance and decreased insulin resistance in obese mice [12]. It is previously known that SPX is an important regulator of cell proliferation in different cell types; for example, it stimulates proliferation in the muscle C2C12 cell line [13] and pancreas INS-1E cells [14], whereas in rat adrenocortical cells it decreases bromodeoxyuridine (BrdU) incorporation [15]. SPX action is strictly connected with activation of signaling kinases pathways, including the upregulation of mitogen-activated kinase (MAP3/1) in mice osteoblasts [16]. In comparison, insulin inhibits the SPX response by activating MAP3/1 and protein kinase B (AKT) pathways in the mouse stomach [17].

Some evidence also indicated a close relationship between SPX and reproduction, by describing its expression in tissues of the hypothalamus–pituitary–gonads (HPG) axis [18–20]. Its presence was observed in the human [21] and fish [18] hypothalamus, where it was increased during fasting [18]. In the fish pituitary, the SPX level was inhibited in the spawning period [19], which indicated its regulation by reproductive hormones. SPX also directly regulates fish pituitary function by decreasing luteinizing hormone (LH) secretion [22] and follicle stimulating hormone (*Fsh*) gene expression [23]. However, data about ovarian expression of SPX are limited and its role in ovarian cell physiology is largely unexplored.

Interestingly, recent studies suggest that SPX may be a novel key player in the pathophysiology of polycystic ovarian syndrome (PCOS); the serum level of SPX is inversely associated with androgens and metabolic profiles in PCOS women [24]. Also, our previous data documented that *Spx* transcript was decreased in the ovary of letrozole-induced PCOS rats [20]. In the pathophysiology of PCOS, insulin resistance and disruptions of the HPG axis were noted, including increased LH secretion [25]. Approximately 50% of women with PCOS are overweight or obese [26], and it is a well-known fact that obesity increases insulin resistance and hyperinsulinemia, which in turn stimulates adipogenesis [26]. Besides, most PCOS patients with hyperandrogenism have defects of steroid secretion that result in abnormal folliculogenesis and failed dominant follicle selection [27], while inhibition of granulosa cells (GC) proliferation is recognized as a key factor that underlies aberrant follicle maturation [28]. All PCOS patients have symptoms such as menstrual irregularity, infertility, miscarriage, and metabolic syndrome. Interestingly, obese women with PCOS have a more severe phenotype than normal weight women with PCOS [29]. Thus, the role of adipokines, to which SPX belongs, has been implicated to play important roles in the pathogenesis of PCOS [30]. Levels of adipokines change significantly in the plasma of PCOS women: concentrations of omentin [31] and adiponectin [32] were decreased, whereas the opposite effect was observed for leptin [33] and visfatin [32], suggesting adipokines as a simple diagnostic criteria of PCOS. In addition, increased levels of vaspin in obesity and PCOS play a role in PCOS pathogenesis, notably protecting from insulin-resistance-induced complications [34].

Thus, we hypothesize that SPX and GALR2/3 are expressed in human ovarian cells and the follicular fluid (FF) of women and that SPX directly regulates GC function. Hence, in the present work, we investigated (i) gene and protein expression of SPX and GALR2/3 in GC of normal weight and obese healthy women and diagnosed with PCOS, its immunolocalization in the human ovary, as well as SPX levels in FF; (ii) the in vitro effect of SPX on GC proliferation and steroidogenesis; and (iii) the molecular mechanism of SPX action by GALR2/3 silencing and pharmacological inhibition of kinases: MAP3/1, AKT, and protein kinase A (PKA), as well as signal transducer and activator of transcription 3 (STAT3).

## Material and methods

### Ethics statement

The study was conducted according to the principles set out in the Declaration of Helsinki. Informed consent was obtained from each participant, and the study protocol was approved by the Institutional Review Board (authorization protocol 2016_075, Ethic Committee of Tours University Hospital, France).

#### Study population

Studied patients were recruited among women at 21–41 years (32 ± 4.7) admitted to the referral Centre for Reproductive Medicine of Tours University Hospital (2011–2022) for infertility requiring in vitro fertilization (IVF) assistance. The ovarian stimulation protocol and IVF, which were the same for all patients included in the study, were described previously [35]. Patients were classified as having PCOS according to the Rotterdam criteria. Hormonal profile: FSH, LH, and testosterone were measured in blood samples collected between the 3rd and 5th days of the cycle by enzyme-linked immunosorbent assay (ELISA) using an Immulite 2500 immunoassay analyzer (Siemens, Munich, Germany), while anti-Müllerian hormone was determined by Eurofins Biomnis (Lyon, France). A transvaginal ovarian ultrasound scan was performed in the early follicular phase to determine the antral follicle number and to assess ovulation. According to body mass index (BMI), PCOS and control/healthy patients were classified as normal weight (BMI 18–25 kg/m^2^) or obese (BMI > 30 kg/m^2^). Characterization of patients is presented in Supplementary Table 1. Samples from a total of 164 patients (41 in each group: normal weight, obese, PCOS normal weight, and PCOS obese) were collected in this study.

#### Collection and processing of biological samples

The FF and primary human luteinized GC were obtained from the cumulus–oocyte (Oo) complexes collected during Oo retrieval as part of the IVF procedure. Fluid collected after punctuation was centrifuged (400 × *g* for 10 min) to separate cell remnants from FF. Then, FF was stored at −80°C for later analyses. Human GC were isolated by 20 min of centrifugation at 400 × *g* on a two-layer discontinuous Percoll (Cytiva) gradient (40, 60%) in 10% fetal bovine serum (FBS) in McCoy (Sigma-Aldrich) medium (Supplementary Table 2) to remove erythrocytes, recovered in McCoy medium, and centrifuged again. Human GC were stored at −80°C for later analyses or immediately cultured.

#### GC in vitro culture and experimental protocol

Human GC were obtained as described above from patients. In addition, we used the KGN steroidogenic human GC-like tumor cell line expressing functional FSH receptors (supplied by Prof Nishi, Kyushu University, Fukuoka, Japan) [36]. KGN were cultured in DMEM (Eurobio Scientific) with 10% FBS and penicillin–streptomycin 1% and human GC in McCoy medium containing FBS 10%, penicillin–streptomycin 1%, apotransferrin 5 mg/L, HEPES 20 mmol/L, 4-androstenedione (A4) 0.1 mol/L, and selenium 20 μg/L. Cells were dispensed into 96-well culture plates and incubated at 37°C 95% O_2_/5% CO_2_. After 24 h, the medium was replaced with fresh medium containing 1% FBS, and cells were starved for 24 h to reduce effect of hormones or growth factors presented in FBS.

*Experiment 1:* to investigate the effect of SPX on GC proliferation after 24 h of starvation, GC were treated with SPX (Phoenix Pharmaceuticals) at doses of 0.1–100 nM [24] for 24, 48, or 72 h. Moreover, the collective effect of insulin-like growth factor type 1 (IGF1; Merck) or FSH (10^−8^ M; Merck) [34] and SPX (1 nM) on GC proliferation was measured by alamarBlue assay. In addition, cells were washed in PBS and stored at −80°C for quantification of proliferating cell nuclear antigen (*PCNA*) mRNA levels or boiled in Laemmli buffer for 4 min for the protein expression of PCNA.

*Experiment 2:* to study the role of SPX in GC steroidogenesis and GALR2/3 expression, cells were treated with SPX at doses of 0.1–100 nM for 48 h in the presence of A4 (0.1 mol/L) and IGF1 or FSH alone or with the addition of SPX (1 nM). Next, the culture medium was collected to investigate progesterone (P4) and estradiol (E2) levels, while cells were washed in PBS and stored at −80°C for mRNA quantification of steroidogenic acute regulatory protein (*STAR*), cytochrome P450 family 11/17 subfamily member A1 (*CYP11A1*, *CYP17A1*), 17β-hydroxysteroid dehydrogenase (*HSD17B*), 3β-hydroxysteroid dehydrogenase (*HSD3B*), and aromatase (*CYP19A1*) or boiled in Laemmli buffer for 4 min for the protein expression measurement of steroidogenic enzymes and GALR2 and GALR3 receptors.

*Experiment 3:* to study the molecular mechanism of SPX action on GC, firstly we analyzed kinase phosphorylation patterns. Briefly, KGN cells were incubated with SPX (1 nM) for 1, 5, 15, 30, 45, and 60 min. Subsequently, cells were washed in PBS and boiled in Laemmli buffer for 4 min for the pMAP3/1/MAP3/1, pAKT/AKT, pSTAT3/STAT3, and pPKA/PKA protein expression.

*Experiment 4:* in the next part of the experiments, cells were pre-treated for 1 h with pharmacological inhibitors of MAP3/1 (PD98059, at a dose of 10 μM; Tocris), AKT (LY294002 at 1 μM; Cell Signaling Technology), or STAT3 (AG490 at 1 μM; Sigma-Aldrich), and PKA (KT570 at 5 ng/mL; Sigma-Aldrich), respectively. The doses of inhibitors were based on our previous papers [38]. Subsequently, SPX at a concentration of 1 nM was added for the next 48 h, and then alamarBlue was added for cell proliferation measurement, or the culture medium was collected for E2 concentration analysis.

*Experiment 5:* finally, we investigated GALR2/3 involvement in SPX action on GC proliferation and steroidogenesis. To validate the efficiency of silencing, KGN were incubated for 24 h in DMEM without FBS and then transfected with GALR2/3 or negative control siRNA (10, 15, and 20 pM; Thermo Fisher Scientific) using lipofectamine RNAiMAX (Thermo Fisher Scientific) according to the manufacturer’s instructions. After an additional 24 h of incubation, GALR2 and GALR3 gene and protein expression were assessed. According to transfection efficiency, we selected a dose of 20 pM for subsequent experiments. Subsequently, after 24 h of incubation with *GALR2* or *GALR3* siRNA, we added SPX (1 nM) for an additional 48 h. Next, alamarBlue (Invitrogen) was added for cell proliferation measurement, or culture medium was collected for E2 concentration analysis.

#### Real-time quantitative polymerase chain reaction

RNA extraction was performed with TRIzol Reagent according to the manufacturer’s procedure. Subsequently, RNA and cDNA quantity were evaluated by measuring absorbance at the 260- and 280-nm wavelengths by spectrophotometry [39]. Reverse transcription was performed using a Promega kit according to the manufacturer’s instructions. Then, specific primers were employed as described (Table 1) following qPCR protocols described previously [39]. The relative mRNA expression levels of the studied genes were determined using the 2^−ΔΔCt^ method.

**Table 1:** Primer sequence employed for real-time quantitative PCR.

Gene	Forward primer 5′ → 3′	Reverse primer 5′ → 3′
*SPX*	GAGGCAGCAACCATCTTA	TTCACCAGTTAAGCAGACT
*GALR2*	ATGGACATCTGCACCTTCGT	GTAGGTCAGGCCGAGAACC
*GALR3*	TTTACGCTGGCTGCTGTCTC	CGGTGCCGTAGTAGCTGAG
*PCNA*	CTCAAGAAGGTGTTGGAGGC	GTAGGTGTCGAAGCCCTCAG
*STAR*	AAACTTACGTGGCTACTCAGCATC	GACCTGGTTGATGCTCTTG
*CYP11A1*	CAGGAGGGGTGGACACGAC	AGGTTGCGTGCCATCTCATAC
*CYP17A1*	GCATCATAGACAACCTGAGCAA	GGGTTTTGTTGGGGAAAATC
*HSD3B*	GCCTTCCAGACCAGAATTGAGAGA	TCCTTCAAGTACAGTCAGCTTGGT
*HSD17B*	GAGACATTCTGGATGAGCC	CGCACAAGTGTACAAGGTAT
*CYP19A1*	GAGAATTCATGCGAGTCTGGA	CATTATGTGGAACATACTTGAGGACT
*PPIA*	ATGCTGGACCCAACACAAAT	TCTTTCACTTTGCCAAACACC
*ACTB*	GTCCCAGTCTTCAACTATAC	ACGGAACCACAGTTATCAT
*GAPDH*	ATGGAAATCCCATCACCATCTT	CGCCCCACTTGATTTTGG

#### Western blot

Cell lysis, western blotting, electrophoresis, and transfer were performed as we described previously [39]. Protein (30 μg) of each sample was used for western blot. Primary and secondary antibodies are described in Supplementary Table 3. As a loading control, beta-actin (ACTB) or alpha-tubulin (TUBA) was used. The WesternBright Quantum HRP substrate (Advansta Inc.) was used to determine the fluorescence signal, which was visualized using the ChemiDoc XRS + System (BioRad,). Densitometry analysis was performed in ImageJ software (US National Institutes of Health) to quantify all visible bands.

#### Enzyme-linked immunosorbent assay

To determine the SPX level in FF and steroids in cultured GC, ELISA kits for SPX (Phoenix Pharmaceuticals), P4, and E2 (DRG Instruments GmbH) were used. The limit of assay sensitivity was 0.11 ng/mL for SPX, 0.045 ng/mL for P4, and 9.714 pg/mL for E2. The inter- and intra-experimental coefficients of variation were, respectively, <10% and <15% for SPX, <9.96% and <6.99% for P4, and <9.39% and <6.81% for E2. Samples were run in duplicate within the same assay. Absorbance was measured at the 450 nm wavelength for SPX or the 405 nm wavelength for steroids using a Varioskan LUX reader (Thermo Fisher Scientific).

#### Immunohistochemistry

Ovary serial sections (Biomax; Supplementary Table 2) from normal weight healthy (non-PCOS) women aged 21 years were used as described previously [39, 40]. Sections were incubated with SPX, GALR2, and GALR3 primary antibodies at a 1:50 dilution followed by the appropriate secondary antibodies (Supplementary Table 3). Negative controls were obtained by replacing the primary antibody with rabbit or goat Immunoglobulins G (Sigma-Aldrich).

#### alamarBlue assay

To determine GC proliferation, the alamarBlue stock solution was aseptically added to wells in amounts equal to 10% of the medium incubation volume. Subsequently, after 2 h of incubation with alamarBlue, the fluorescence was determined at the 530- and 590-nm wavelengths using a Varioskan LUX reader (Thermo Fisher Scientific).

### Statistical analysis

Results are shown as mean ± SEM. One-way and two-way ANOVA followed by post hoc tests were used. Results were not adjusted for age or any other factor. All data were also tested for the assumptions of normality (Shapiro–Wilk test) and homogeneity of variances (Levene test). Statistical analysis was performed using GraphPad Prism 8 (PRISM) software, significance was checked at the level *P* <0.05, and detailed *P*-values were added in Supplementary Table 4. All in vitro experiments were repeated a minimum of three times.

## Results

### Gene and protein expression of SPX and GALR2/3 in the human ovarian cells and SPX levels in FF

**Figure 1:**
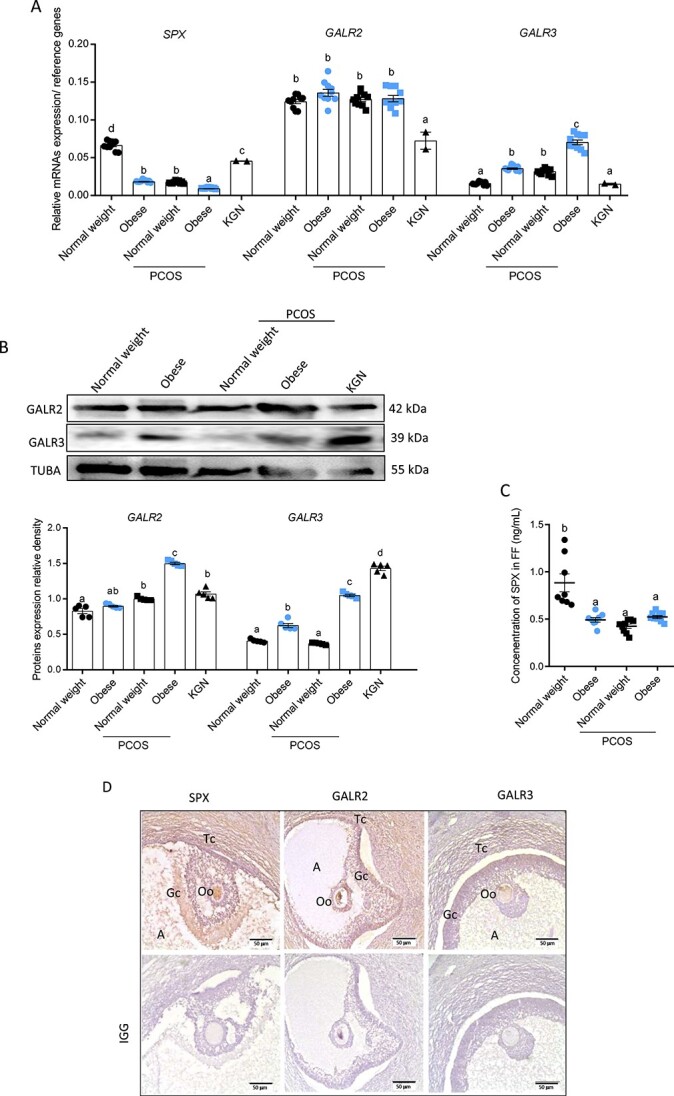
Comparison of SPX, GALR2, and GALR3 mRNA (**A**) and protein (**B**) expression in GC and its level in FF (**C**) collected from normal weight, obese, PCOS normal weight, and PCOS obese women (*n* = 10) and KGN as a control (*n* = 2). SPX, GALR2, and GALR3 immunolocalization (**D**) in human ovary (*n* = 3). Representative blots and immunohistochemistry photos at ×20 are shown. Data are plotted as the mean ± SEM. One-way ANOVA, followed by post hoc tests, was used for statistical analysis (GraphPad Prism 8). Significance between groups is indicated by different letters (*P* < 0.05). IGG: immunoglobulin G, A: antrum, TUBA: alpha-tubulin, Tc: theca, Cc: cumulus cells, Oo: oocyte, Gc: granulosa cells.

We noted that *SPX* gene expression was decreased in GC of obese, PCOS normal weight, and PCOS obese subjects compared with normal weight women by 3.7, 4.13, and 7.33 folds, respectively (Figure 1A, Supplementary Table 4, *P* < 0.05). The transcription level of *GALR2* was stable between groups. Gene expression of *GALR3* was increased in GC of obese, PCOS normal weight, and PCOS obese compared to normal weight by 1.66, 2.25, and 1.56 folds, respectively (Figure 1A, *P* < 0.05). At the protein level, we observed that GALR2 expression was elevated in GC of PCOS normal weight and obese compared to normal weight women. Expression of GALR3 was increased in GC of obese and PCOS obese compared to normal weight women (Figure 1B, *P* < 0.05). There is no available anti-SPX antibody for western blot; therefore, its level was not analyzed.

We observed decreased levels of SPX in FF from the obese group at 0.49 ± 0.02 ng/mL, PCOS normal weight at 0.42 ± 0.02 ng/mL, and obese PCOS at 0.52 ± 0.02 ng/mL compared to 0.88 ± 0.07 ng/mL in normal weight women (Figure 1C, *P* < 0.05). Moreover, we detected an SPX signal and both receptors GALR2/3 in GC, theca cells (Tc), Oo, and surrounding cumulus cells (Cc; Figure 1D).

The decreased transcript level of *SPX* and *GALR2* was described also in KGN cell line compared with normal weight controls by 1.75 and 1.66 folds, respectively, while no change in GC of normal weight women and KGN was observed (Figure 1A, *P* < 0.05). Both GALR2 and GALR3 protein expression was elevated compared to normal weight control (Figure 1B, *P* < 0.05).

### Effect of SPX on basal and IGF1- and FSH-induced proliferation in human GC

**Figure 2:**
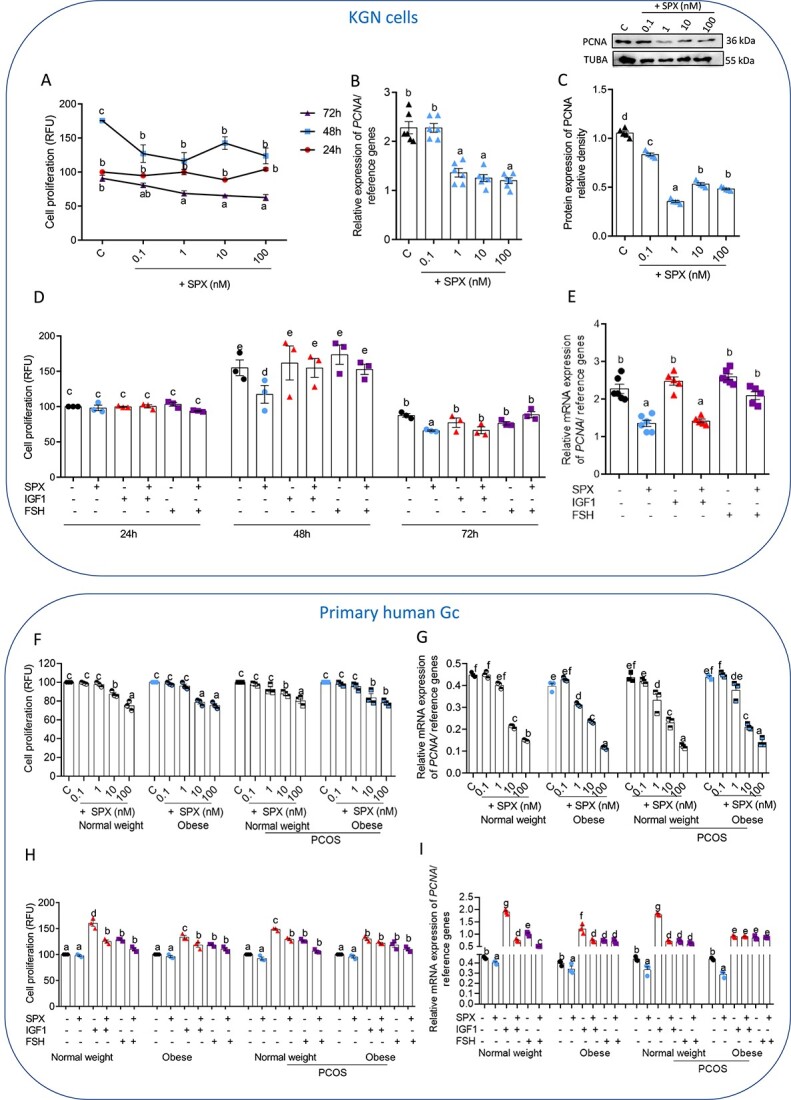
Dose-dependent effect of SPX added alone or in combination with IGF1, or FSH on KGN cell proliferation (**A**, **D**) and PCNA mRNA (**B**, **E**) and protein level (**C**) (*n* = 6) as well as the dose-dependent effect of SPX added alone or in combination with IGF1, or FSH on human GC proliferation (**F**, **H**) and PCNA transcript (**G**, **I**) (*n* = 3). Representative blots are shown. Data are plotted as the mean ± SEM. One-way ANOVA was used to compare SPX effect on PCNA level in KGN cells, while two-way ANOVA was used for other analyses, followed by post hoc tests (GraphPad Prism 8). Significance between control and treatments is indicated by different letters (*P* < 0.05). C: control, TUBA: alpha-tubulin, PCNA: proliferating cells nuclear antigen.

As shown in Figure 2A, SPX at all investigated doses decreased KGN cell proliferation after 48 h and at doses of 1, 10, and 100 nM after 72 h of cell culture. The latter observation was confirmed on the *PCNA* mRNA level, which was inhibited by SPX at 1, 10, and 100 nM (Figure 2B, Supplementary Table 4, *P* < 0.05). Besides, SPX at all investigated doses inhibited PCNA protein levels in KGN cells (Figure 2C). As we noted, SPX had no effect on IGF1- and FSH-induced proliferation of KGN cells whatever the time of stimulation (Figure 2D), but reduced after 48 h incubation of IGF1-induced *PCNA* gene expression (Figure 2E, *P <* 0.05).

In human primary GC, we showed that SPX at 10 and 100 nM decreased cell proliferation significantly in all investigated groups after 24 h (Figure 2F, *P* < 0.05). In addition, SPX decreased *PCNA* transcript levels at doses of 10 and 100 nM in GC of normal weight and PCOS obese women and at doses of 1, 10, and 100 nM in obese and PCOS normal weight women (Figure 2G, *P* < 0.05). As shown in Figure 2H, SPX addition had negative effects on IGF1-induced proliferation in all groups except for GC of PCOS obese women (Figure 2H, *P* < 0.05). It was confirmed by analysis of *PCNA* transcript levels. In addition, we observed no effect of SPX on FSH-induced cell proliferation, whereas in GC of normal weight women, we noted a decrease in *PCNA* expression after SPX with FSH (Figure 2I, *P <* 0.05).

#### Effect of SPX on basal and IGF1- and FSH-induced P4 and E2 secretion by human GC

**Figure 3:**
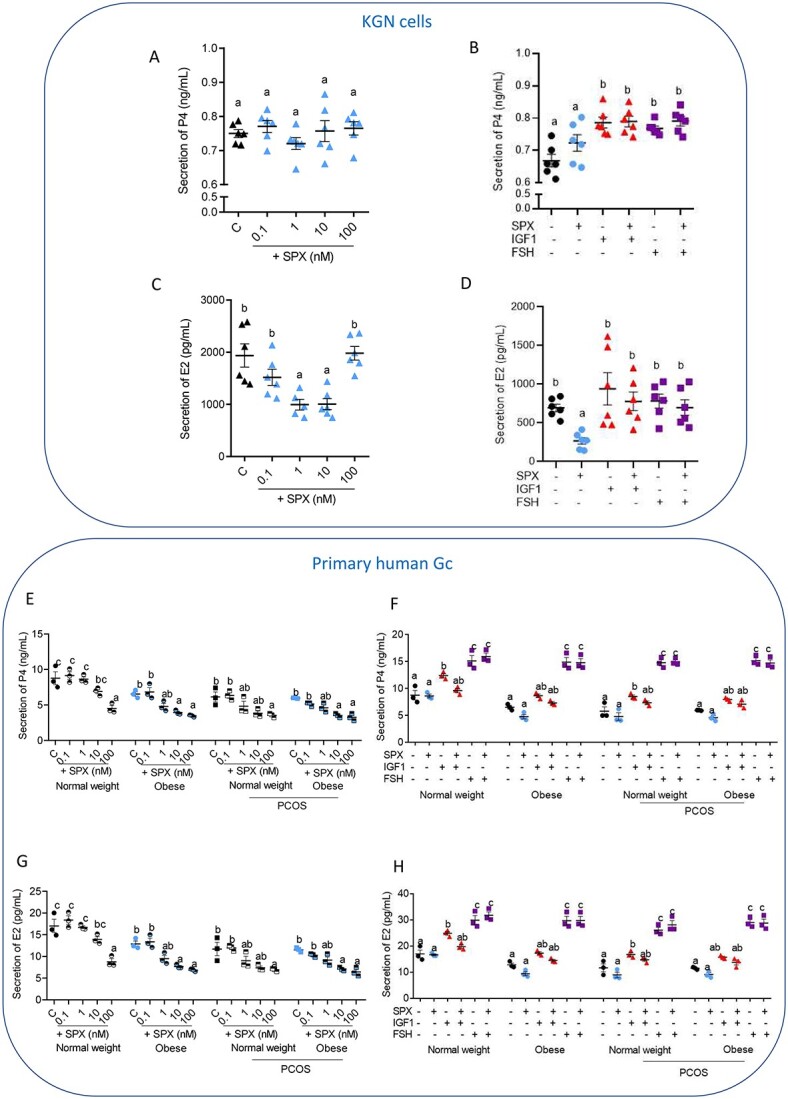
Dose-dependent effect of SPX added alone or in combination with IGF1, or FSH on P4 (**A**, **B**) and E2 (**C**, **D**) secretion in KGN cells (*n* = 6), as well as dose-dependent effect of SPX added alone or in combination with IGF1, or FSH on P4 (**E**, **F**) and E2 (**G**, **H**) secretion by primary human GC (*n* = 3). Data are plotted as the mean ± SEM. One-way ANOVA was used to compare SPX effect on P4 and E2 level in KGN culture medium, while two-way ANOVA was used for the rest of the analysis, followed by post hoc tests (GraphPad Prism 8). Significance between control and treatments is indicated by different letters (*P* < 0.05). C: control P4: progesterone, E2: estradiol.

As shown in Figure 3A, SPX had no effect on P4 secretion by KGN cells (Supplementary Table 4). As expected, treatment with either IGF1 or FSH increased the P4 level in KGN cells; however, no additional effect was observed when we combined IGF1 and FSH treatment with SPX (Figure 3B, *P* < 0.05). Interestingly, SPX at doses of 1 and 10 nM decreased E2 secretion (Figure 3C, *P* < 0.05), while no effect on IGF1- and FSH-induced E2 secretion was observed (Figure 3D).

In primary human GC, we observed that SPX at a dose of 100 nM decreased P4 secretion by GC of normal weight women, at doses 10 and 100 nM in obese women, at dose 100 nM in normal weight women with PCOS, and at 10 and 100 nM in obese women with PCOS (Figure 3E, *P* < 0.05). Furthermore, we noted an inhibitory effect of SPX on E2 secretion by GC in women of all studied groups: at 100 nM in the normal weight group, and at 10 and 100 nM in the obese, PCOS normal weight, and PCOS obese groups (Figure 3G, *P* < 0.05). Moreover, no effect of SPX was induced in response to both IGF1 and FSH on P4 (Figure 3F) and E2 (Figure 3H) steroid secretion by GC of all investigated groups.

#### Effect of SPX on basal and IGF1- and FSH-induced expression of STAR protein and steroidogenic enzymes in human GC

**Figure 4:**
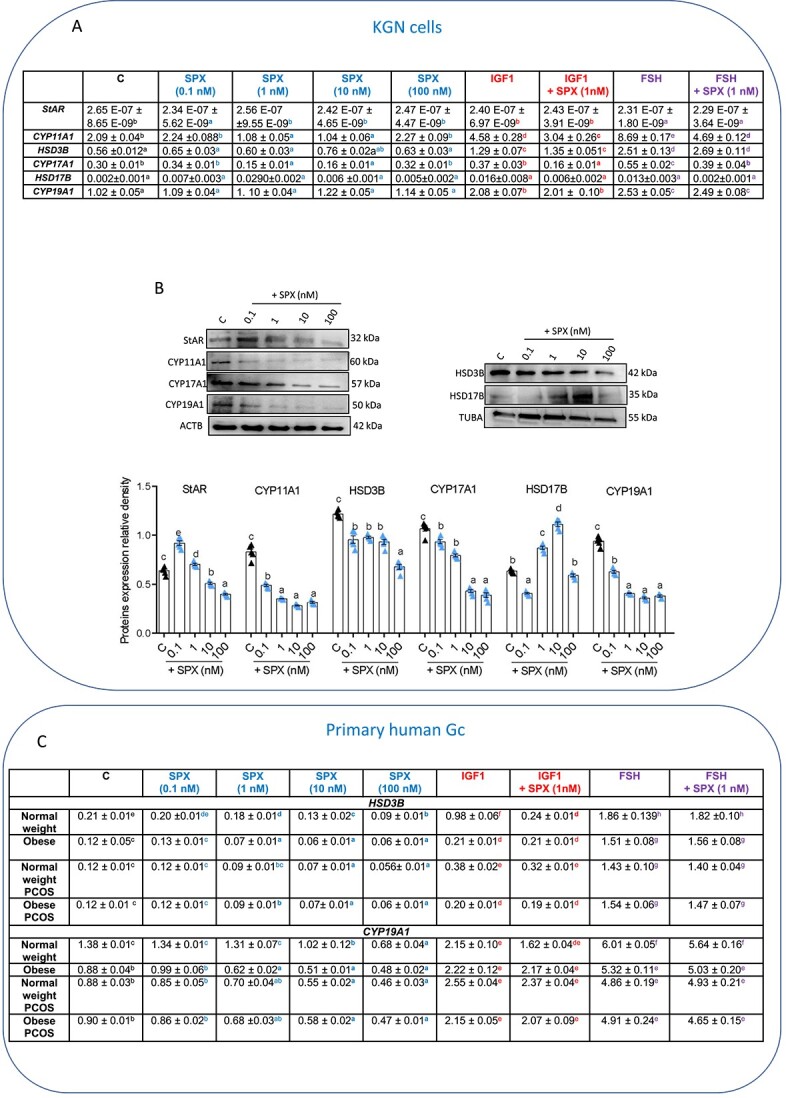
Dose-dependent effect of SPX added alone or in combination with IGF1, or FSH on STAR protein and steroidogenic enzymes expression in KGN cells (**A**, **B**) (*n* = 6) and primary human GC (**C**) (*n* = 3). Representative blots are shown. Data are plotted as the mean ± SEM. One-way ANOVA was used to compare SPX effect on enzymes level in KGN cells, while two-way ANOVA for was used for the rest of the analysis, followed by post hoc tests (GraphPad Prism 8). Significance between control and treatments is indicated by different letters (*P* < 0.05). C: control, CYP11A1, CYP17A1: cytochrome P450 family 11/17 subfamily member A1, HSD17B: 17β-hydroxysteroid dehydrogenase, HSD3B: 3β-hydroxysteroid dehydrogenase, CYP19A1: aromatase, TUBA: alpha-tubulin, ACTB: beta-actin.

We noted that in KGN cells, SPX decreased the gene expression of *STAR* significantly at 0.1 nM, and that of *CYP11A1* and *CYP17A1* at doses 1 and 10 nM (Figure 4A, Supplementary Table 4, *P* < 0.05), while no effect on transcript levels of *HSD3B*, *HSD17B*, and *CYP19A1* was demonstrated. Moreover, we observed that SPX (1 nM) decreased both IGF1- and FSH-induced *CYP11A1* and *CYP17A1* mRNA expression (Figure 4A, *P* < 0.05).

As shown in Figure 4B, we observed that SPX at doses 0.1 and 1 nM increased, while at 10 and 100 nM decreased protein expression of STAR and at all investigated doses decreased expression of CYP11A1, HSD3B, CYP17A1, and CYP19A1 protein. Interestingly, SPX at dose 0.1 nM decreased, while at 1 and 10 nM it increased HSD17B protein levels.

In primary human GC, we observed that SPX at 1, 10, and 100 nM decreased *HSD3B* transcript levels in GC of the normal weight, obese, and PCOS obese groups and at doses of 10 and 100 nM in the PCOS normal weight group (Figure 4C, *P* < 0.05). SPX had an inhibitory effect on *CYP19A1* expression at 10 and 100 nM in the normal weight, PCOS normal weight, and PCOS obese groups and at doses of 1, 10, and 100 nM in GC of obese women. Furthermore, in GC of normal weight women, we noted that SPX decreased the IGF-induced *HSD3B* level, whereas it had no effect in the response to FSH (Figure 4C, *P* < 0.05).

#### Effect of SPX on GALR2/3 expression and phosphorylation of kinases in human GC

**Figure 5:**
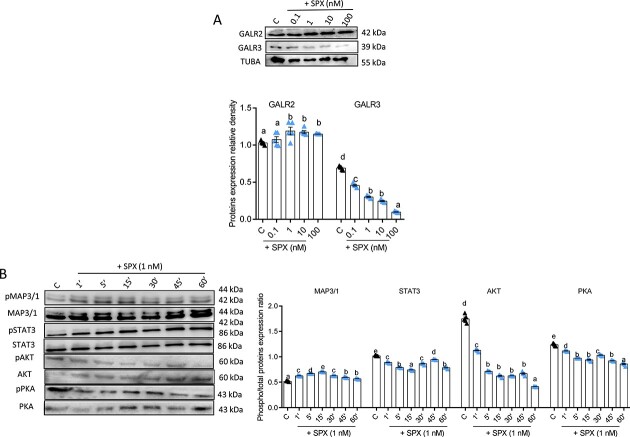
Dose-dependent effect of SPX on GALR2 and GALR3 expression (**A**), as well as time-dependent effect on MAP3/1, PKA, STAT3, and protein kinase B (AKT) phosphorylation (**B**) (*n* = 5). Representative blots are shown. Data are plotted as the mean ± SEM. One-way ANOVA followed by post hoc tests was used (GraphPad Prism 8). Significance between control and treatments is indicated by different letters (*P* < 0.05). C: control, MAP3/1: mitogen activated kinase, PKA: protein kinase A, STAT3: signal transducer and activator of transcription 3, AKT: protein kinase B.

We showed that SPX at 1, 10, and 100 nM elevated GALR2 protein levels, whereas all investigated doses inhibited GALR3 expression (Figure 5A, Supplementary Table 4, *P* < 0.05). We noted that SPX at 1 nM modulated phosphorylation of the following kinase pathways in a time-dependent manner: at 1, 5, 15, 30, 45, and 60 min, it stimulated the phosphorylation of MAP3/1 and reduced the phosphorylation of PKA, STAT3, and AKT (Figure 5B, *P* < 0.05).

#### Involvement of GALR2/3 and MAP3/1, PKA, STAT3, and AKT in SPX action on proliferation and E2 secretion by human GC

**Figure 6:**
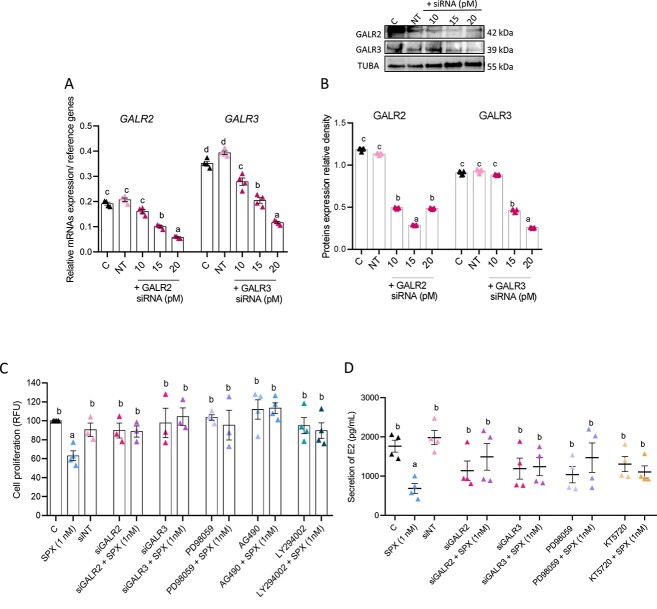
Involvement of GALR2 and GALR3, as well as MAP3/1, PKA, STAT3, and protein kinase B (AKT) in SPX action on KGN cell proliferation and E2 secretion (*n* = 4). GALR2 and GALR3 receptors silencing on mRNA (**A**) and protein (**B**) level and finally involvement of galanin receptors and several kinases in SPX-mediated proliferation (**C**) and E2 secretion (**D**) are presented. Representative blots are shown. Data are plotted as the mean ± SEM. One-way ANOVA followed by post hoc tests was used (GraphPad Prism 8). Significance between control and treatments is indicated by different letters (*P* < 0.05). C: control, NT: non-target, TUBA- alpha-tubulin.

Next, the efficiency of GALR2/3 silencing was checked: on the mRNA level, siRNA for *GALR2* at 15 and 20 pM decreased its levels to 57 and 31%, while *GALR3* siRNA at 10, 15, and 20 pM decreased its transcript levels to 84, 52, and 30%, relative to the control (100%) (Figure 6A, Supplementary Table 4, *P* < 0.05). This observation was partly confirmed on the protein level except at a dose of 10 pM (Figure 6B, *P* < 0.05). Finally, both siRNA and pharmacological inhibitors PD98059, AG490, and LY294002 added along with SPX reduced the SPX inhibitory effect on GC proliferation (Figure 6C, *P* < 0.05), while both siRNA and inhibitors PD98059 and KT5720 added along with SPX reduced the effect of SPX on E2 secretion (Figure 6D, *P* < 0.05). We also observed that both siRNA and kinase inhibitors added alone had no effect on GC proliferation or E2 secretion.

## Discussion

The present study was the first to demonstrate expression of the SPX/GALR2/GALR3 system in human GC. We observed decreased levels of SPX in GC and FF of obese and PCOS women, in contrast to the expression of galanin receptors. In addition, we confirmed our hypothesis that SPX affects human GC function and we noted a negative in vitro effect of SPX on GC physiology including steroidogenesis and proliferation via activation of GALR2/3 receptors and kinases MAP3/1, PKA, STAT3, and AKT, which indicate that SPX may be a novel modulator of GC physiology and closely linked with PCOS.

Our data showed, for the first time, decreased expression of the SPX transcript in human GC collected from obese women and those diagnosed with PCOS. Thus, we can confirm that ovarian cells are able to produce SPX and change its level in FF, as was noted previously for other adipokines such as omentin [39]. Our data are in agreement with the literature: results of Respekta et al. [20] which showed that SPX expression was decreased in the ovary of a letrozole-induced PCOS rat model. Other studies confirmed that SPX levels in mouse stomachs decreased in obesity and elevated insulin resistance, closely related to PCOS [17]. It is previously clearly indicated that SPX levels in serum may be a novel predictor of the pathogenesis of PCOS. Guler et al. [24] observed significantly lower levels of SPX in human plasma of PCOS and obese patients. Our results for SPX levels in FF confirmed those of a previous study. Moreover, we observed that expression of the *GALR3* transcript was increased, but the *GALR2* level was stable in GC of obese and PCOS women, while on the protein level, the expression of both receptors was elevated. Differences between the gene and protein levels may be explained by complex and diverse post-transcriptional mechanisms involved in converting mRNA to protein not yet sufficiently defined to be able to calculate protein concentrations from mRNA [41]. Our observation on GALR2/3 expression in human GC was partly confirmed by the literature. Expression of the *Galr2/3* transcript increased in mouse subcutaneous adipose tissue [42] and rat ovary in response to a high-fat diet [43]. In addition, acute treatment with palmitate, a dietary saturated fatty acid, stimulated *Galr2/3* expression in hypothalamic neurons of mice [44]. Moreover, the expression of galanin receptors is probably changed by galanin, of which the serum level is elevated in obesity and PCOS [45]. In addition, higher levels of testosterone in PCOS patients were strongly correlated with increased *Galr2* expression in proopiomelanocortin rat neurons [46], while E2 stimulated *Galr3* expression in the pituitary of female rats [47].

We observed immunolocalization of SPX and GALR2/3 in GC and Tc, as well as Oo and Cc, which is in agreement with the previous data that describe its expression in rat ovaries. In rats, SPX immunoreaction was mainly cytoplasmic in thecal, luteal, and interstitial cells of the ovary [3]. Nevertheless, Respekta et al. [20] showed nuclear-cytoplasmic localization of SPX in GC, whereas localization was nuclear in the theca, with no differences between control and PCOS rats. Ovarian expression of different adipokines and their receptors has been widely described in ovarian cells of obese and PCOS women, indicating their potential therapeutic or marker roles. Higher levels of vaspin and its receptor were observed in GC of obese women, where vaspin regulates GC steroidogenesis and proliferation [34]. Moreover, chemerin levels were elevated in both FF and GC samples from patients with PCOS, and its treatment attenuated insulin-stimulated glucose uptake by decreasing phosphorylation of insulin receptor substrate [48]. Thus, expression of SPX as a ligand and its receptors GALR2/3 in the GC, as well as the described role of adipokines in PCOS, suggests that SPX may also be an interesting player in GC physiology and future target for treatment intervention. Therefore, we can speculate not only endocrine but also autocrine and/or paracrine effects of SPX in GC.

The proliferation of GC is an important process in ovarian physiology necessary for proper folliculogenesis and even Oo growth [49]. During follicle formation, the morphological and physiological properties of GC change and are connected to cells’ capacity for continuous division. Moreover, GC provides nutrients and growth regulators for the development of Oo, which are essential for follicular development and ovulation [28]. Therefore, delays in proliferation can inhibit follicular development, which likely plays an important role in the pathogenesis of PCOS [49]. In our study, we showed that SPX decreased GC proliferation and PCNA levels in human GC. The prolonged exposure of adrenocortical cell primary cultures to SPX resulted in a notable decrease in BrdU incorporation [15], which indicated its inhibitory effect on endocrine tissue. However, SPX stimulated proliferation in the C2C12 muscle cell line [13] and had no effect on 3T3L1 adipocytes [11]. Interestingly, in human GC, SPX decreased proliferation at a higher dose compared to KGN, and an inhibitory effect was observed at 10 nM. To explain our observations, it should be mentioned that galanin receptors have the ability to bind a variety of ligands, including galanin [4]. Thus, we can hypothesize that SPX receptors in human GC may be blocked by galanin, but to our knowledge, the galanin level in human GC and KGN was never compared. For example, higher expression of chemerin receptor chemokine-like receptor 1 was noted in primary human GC than in KGN [50]. In our experiments, we tested the role of SPX in IGF1 and FSH-stimulated proliferation in human GC because both IGF1 and FSH are well known to play a key role in the development of antral follicles and steroid synthesis, thus decreasing in its synthesis and leading to fertility disturbances [51]. We observed a lack of additional effects of SPX on IGF1 or FSH-stimulated proliferation in KGN, but we noted a significant decrease in IGF1-stimulated PCNA expression. Interestingly, SPX decreased FSH secretion in tilapia [23], while the insulin rise caused by glucose uptake presumably originated from the pancreas and may serve as negative feedback to inhibit the SPX response in mice [17]; however, future investigations are needed to understand the interaction between SPX and FSH or IGF1 in human GC. Moreover, differences between SPX-IGF1/FSH interactions in cell proliferation and the PCNA level were probably dependent on the test used: PCNA is a marker of proliferation, whereas alamarBlue measures cell viability, which may be regulated by different kinase pathways, but this requires future tests. For example, resistin mediated luteal cell proliferation via AKT kinase pathways, showing no differences in PCNA protein levels while the stimulatory effect on viability remained unchanged [52]. Interestingly, SPX did not inhibit IGF1-induced proliferation in the GC of the obese PCOS group, which was observed in GC of obese and PCOS normal weight groups. There is a possible explanation for the observed differences: PCOS is often associated with hyperinsulinemia and peripheral insulin resistance; moreover, it is well established that IGF1 acts similarly to insulin. In GC isolated from the ovaries of women with PCOS, insulin action on glucose metabolism is significantly decreased [53], so the differences are probably linked to differing responses to insulin and/or connected with insulin resistance in obese PCOS GC, but this needs further study. Nevertheless, SPX could antagonize the action of IGF1, which plays a key role in the development of ovarian follicles. Besides, as we showed, SPX has no effect on FSH-stimulated proliferation in primary human GC, whereas in GC of normal weight women, we noted a decrease in PCNA expression after SPX with FSH, which is similar to discrepancies observed in KGN for IGF1 and SPX. Similarly, in anovulatory women, galanin did not cause significant increases in LH and FSH levels in serum [54]; taking into consideration the similarities in these two ligands to galanin receptors, it is a possible explanation for the obtained data. A similar phenomenon was observed previously for visfatin in human GC, where no effect on FSH-stimulated proliferation was noted [55].

In the next step, we investigated the effect of SPX on steroidogenesis because proper synthesis of steroid hormones is crucial for follicle growth, ovulation, corpus luteum formation, and hormonal homeostasis in the body [56]. Our data showed that SPX decreased E2 secretion in KGN, and both P4 and E2 in primary human GC in all investigated groups, so the effect of SPX is independent on hormonal and metabolic conditions. As was noted previously, E2 is also a strong mitogenic factor in ovarian follicles [56], thus by inhibiting E2 synthesis, SPX may lead to decreased GC proliferation. The effect of SPX on basal P4 secretion in KGN is statistically irrelevant, but steroid synthesis is dependent on numerous steroidogenic enzyme activities or other factors, including hormones and growth factors [56], which need future studies. On the other hand, this kind of phenomenon has been demonstrated: no effect of visfatin on basal steroid production was noted in primary human GC, whereas visfatin treatment increased P4 and E2 secretion in KGN cells [55]. In this study, we noted that SPX had no effect on IGF-induced P4 and E2 secretion, and it should be explained by the interaction of hormones like E2, insulin, or androgens, the levels of which changed in PCOS and obesity [57]. In human GC, different adipokines, such as adiponectin, visfatin, omentin, and apelin, enhanced IGF1-induced E2 synthesis, while resistin and chemerin seemed rather to act as negative regulators of steroidogenesis [58], so the effect is linked more with properties of the studied adipokine than its levels in serum or FF, which differ between obesity and PCOS. No effect on FSH-stimulated steroidogenesis was observed in our study. Similar to our observation, human recombinant adiponectin had no effect on steroid production in the presence or absence of FSH in rat GC [59], whereas visfatin significantly increased IGF1 but not FSH-induced steroid secretion in human GC and KGN [55]; future studies on the effect of SPX on phosphorylation of IGF and FSH receptors are needed to explain these differences. We confirmed the effects of steroids on *HSD3B* and *CYP19A1* mRNA expression in human GC, whereas in a KGN model, SPX decreased *STAR*, *CYP11A1*, and *CYP17A1*, with no effect on HSD3B, *HSD17B*, and *CYP19A1* transcripts. As is well known, HSD17B participates in A4 conversion to testosterone, so its overexpression may be linked with addition of A4 to the medium, which is necessary for E2 synthesis. An increased A4 concentration is connected with PCOS, in which we observed a decreased SPX level [24]. An additional hypothesis that could explain these results is that SPX could modulate the activity of steroid enzymes, but this requires further investigation. Nevertheless, it has demonstrated differences between enzyme activity and its expression; for example, ciprofibrate decreases HSD3B activity without affecting either HSD3B protein or mRNA expression in rat testis [60]. Interestingly, in our study, we did not observe a clear, positive effect of IGF1 or FSH on E2 secretion or cell proliferation in the KGN line. Differences between the primary GC and the KGN cell line may depend on the different levels of receptor expression for these hormones, the dose used, or post-transcription modifications because we observed a stimulatory effect of both IGF1 and FSH on the expression of *CYP19A1* mRNA in KGN. In addition, as previously shown, primary human GC respond more strongly to IGF1 or FSH in P4 secretion than KGN cells [50]. There are no additional data on the effect of SPX on steroidogenesis in any species, but SPX has a harmful effect on endocrine activity in pituitary cells: in fish, it decreases LH and FSH synthesis [23], and thus it may partly inhibit ovarian steroidogenic function. Other studies also confirmed that insulin secretion from isolated islets was reduced by SPX in rats [14], which indicated its negative global effect on the endocrine system.

It is previously known that SPX participates in regulation of body energy metabolism via activation of galanin receptors, improving lipolysis and fatty acid oxidation via GALR2 signaling in mouse liver [61] and inhibiting adipogenesis and down-regulating mRNA expression of pro-adipogenic genes via GALR2 and GALR3 in human and mouse adipocytes [11]. Also, its effect on proliferation is connected with GALR2/3 activation in C2C12 cells [13]. In our study, we observed that SPX increased GALR2 and decreased GALR3 protein expression. There are several explanations for the inhibition of GALR3 protein: the level of the receptors may be influenced by other ligands, such as galanin, that were not investigated in our study. Interestingly, as shown previously in 3T3L1 adipocytes, galanin increases GALR3 expression with no effect on GALR2 [42]. Decreased GALR3 levels may also be connected with its internalization in response to an elevated SPX level. This phenomenon is well described in the literature: at high plasma concentrations, the number of surface receptors for insulin is gradually reduced by the accelerated rate of receptor internalization and degradation [62]. In addition, higher doses of leptin in serum decreased the mRNA expression of the leptin receptor (*Lepr*) in mouse liver [63], and vaspin decreased GRP78 expression in porcine ovary [64]. Therefore, the lack of effect of SPX on E2 secretion at higher doses may be related to the internalization of GALR3 under the influence of SPX. Thus, taking into account the significant decrease in the expression of this receptor under the influence of SPX, it seems to have a special role in its negative impact on the proliferation and secretion of E2, as previously shown similarly for vaspin, whose receptor mediated the regulation of many processes, including ovarian steroidogenesis [64]. Moreover, it is well described that phosphorylation of kinase pathways is a rapid response to changing environments, as well as in the reproductive system including ovarian follicles, where they participate in processes such as steroidogenesis or Oo maturation. It is previously known that SPX upregulates MAP3/1 in mouse osteoblasts [16]. In our study, we showed that SPX stimulates MAP3/1 phosphorylation but inhibits STAT3, PKA, and AKT. Moreover, we noted that the inhibitory effect of SPX on KGN proliferation was linked to activation of GALR2/3 receptors, as well as MAP3/1, AKT, and STAT3 kinase, while E2 synthesis was linked to both receptors, MAP3/1 and PKA activation. Inhibition of PKA was previously described as connected with decreased P4 synthesis by porcine corpus luteum [65], thus inhibition of phosphorylation may be an answer to negative SPX action on proliferation and E2 synthesis. Similarly, Roche [66] and Maillard [67] showed that other adipokines like apelin and adiponectin increased steroidogenesis by the MAP3/1 pathway in human and bovine ovarian cells, whereas chemerin decreased IGF1-induced steroidogenesis by inhibiting MAP3/1 phosphorylation and proliferation due to the inhibition of the AKT pathway in human GC [50].

In summary, our data first showed decreased SPX levels in GC and FF of PCOS and obese women. In addition, SPX negatively influenced GC function by inhibiting cell proliferation via GALR2/3 and MAP3/1, STAT3, and AKT, and E2 secretion via GALR2/3 MAP3/1, and PKA, which is a feature of PCOS connected with inhibited ovulation and changes in hormones homeostasis. In light of these findings, SPX appears to be a novel modulator of GC physiology and could represent a relevant player in PCOS pathogenesis. However, further studies are needed to characterize the role of SPX in the whole ovary because, as previously described for vaspin, its effect in the porcine ovarian follicles on kinase phosphorylation varies depending on whether it was cultured in co-cultures of GC and Tc or in monocultures [64]. In addition, to confirm our results in the future, it would be necessary to silence SPX expression in GC and observe if this increases cell proliferation and E2 secretion. Nevertheless, adipokine silencing often leads to infertility, as showed previously that female adiponectin null mice displayed impaired fertility, reduced retrieval of Oo, disrupted the estrous cycle, elevated number of atretic follicles, and impaired late folliculogenesis, while adiponectin itself has a positive effect on ovarian function [68]. Thus, future studies are needed to more precisely describe SPX function in the human ovary.

## Supplementary Material

Supplementary_Table_1_ioad108

Supplementary_Table_2_ioad108

Supplementary_Table_3_ioad108

Supplementary_Table_4_ioad108

## Data Availability

The data underlying this article will be shared on reasonable request to the corresponding author.

## References

[1] Mirabeau O, Perlas E, Severini C, Audero E, Gascuel O, Possenti R, Birney E, Rosenthal N, Gross C. Identification of novel peptide hormones in the human proteome by hidden Markov model screening. Genome Res 2007; 17:320–327. 17284679 10.1101/gr.5755407 PMC1800923

[2] Wong MKH, He M, Sze KH, Huang T, Ko WKW, Bian ZX, Wong AOL. Wong AOL. Mouse Spexin:SLive_RefAppend (I) NMR solution structure, docking models for receptor binding, and histological expression at tissue level. Front Endocrinol 2021; 12:e681646. 10.3389/fendo.2021.681646 PMC8285161 34276561

[3] Porzionato A, Rucinski M, Macchi V, Stecco C, Malendowicz LK, De Caro R. Spexin expression in normal rat tissues. J Histochem Cytochem 2010; 58:825–837. 20530460 10.1369/jhc.2010.956300 PMC2924798

[4] Kim DK, Yun S, Son GH, Hwang JI, Park CR, Kim JI, Kim K, Vaudry H, Seong JY. Coevolution of the spexin/galanin/kisspeptin family: spexin activates galanin receptor type II and III. Endocrinology 2014; 155:1864–1873. 24517231 10.1210/en.2013-2106

[5] Lin CY, Zhang M, Huang T, Yang LL, Fu HB, Zhao L, Zhong LL, Mu HX, Shi XK, Leung CF, Fan BM, Jiang M, et al. Spexin enhances bowel movement through activating L-type voltage-dependent calcium channel via galanin receptor 2 in mice. Sci Rep 2015; 5:e12095. 10.1038/srep12095. PMC4498193 26160593

[6] Lin CY, Zhao L, Huang T, Lu L, Khan M, Liu J, Zhong LLD, Cai ZW, Fan BM, Wong AOL, Bian ZX. Spexin acts as novel regulator for bile acid synthesis. Front Physiol 2018; 9:e378. 10.3389/fphys.2018.00378 PMC5902714 29692737

[7] Ma A, Bai J, He M, Wong AOL. Spexin as a neuroendocrine signal with emerging functions. Gen Comp Endocrinol 2018; 265:90–96. 29355530 10.1016/j.ygcen.2018.01.015

[8] Kumar S, Hossain MJ, Javed A, Kullo IJ, Balagopal PB. Relationship of circulating spexin with markers of cardiovascular disease: a pilot study in adolescents with obesity. Pediatr Obes 2018; 13:374–380. 29045048 10.1111/ijpo.12249 PMC5906205

[9] Walewski JL, Ge F, Lobdell H 4th, Levin N, Schwartz GJ, Vasselli JR, Pomp A, Dakin G, Berk PD. Spexin is a novel human peptide that reduces adipocyte uptake of long chain fatty acids and causes weight loss in rodents with diet-induced obesity. Obesity 2014; 22:1643–1652. 24550067 10.1002/oby.20725 PMC4077920

[10] Wong MKH, Chen Y, He M, Lin C, Bian Z, Wong AOL. Mouse spexin: (II) functional role as a satiety factor inhibiting food intake by regulatory actions within the hypothalamus. Front Endocrinol 2021; 12:e681647. 10.3389/fendo.2021.681647 PMC8283969 34276562

[11] Kolodziejski PA, Pruszynska-Oszmalek E, Micker M, Skrzypski M, Wojciechowicz T, Szwarckopf P, Skieresz-Szewczyk K, Nowak KW, Strowski MZ. Spexin: a novel regulator of adipogenesis and fat tissue metabolism. Biochim Biophys Acta Mol Cell Biol Lipids 2018; 1863:1228–1236. 30305242 10.1016/j.bbalip.2018.08.001

[12] Ge JF, Walewski JL, Anglade D, Berk PD. Regulation of hepatocellular fatty acid uptake in mouse models of fatty liver disease with and without functional leptin signaling: roles of NfKB and SREBP-1C and the effects of spexin. Semin Liver Dis 2016; 36:360–372. 27997977 10.1055/s-0036-1597248

[13] Leciejewska N, Pruszyńska-Oszmałek E, Mielnik K, Głowacki M, Lehmann TP, Sassek M, Gawęda B, Szczepankiewicz D, Nowak KW, Kołodziejski PA. Spexin promotes the proliferation and differentiation of C2C12 cells in vitro-the effect of exercise on SPX and SPX receptor expression in skeletal muscle in vivo. Genes 2021; 13:e81. 10.3390/genes13010081. PMC8774514 35052420

[14] Sassek M, Kolodziejski PA, Strowski MZ, Nogowski L, Nowak KW, Mackowiak P. Spexin modulates functions of rat endocrine pancreatic cells. Pancreas 2018; 47:904–909. 29912854 10.1097/MPA.0000000000001083

[15] Rucinski M, Porzionato A, Ziolkowska A, Szyszka M, Macchi V, De Caro R, Malendowicz LK. Expression of the spexin gene in the rat adrenal gland and evidences suggesting that spexin inhibits adrenocortical cell proliferation. Peptides 2010; 31:676–682. 20045034 10.1016/j.peptides.2009.12.025

[16] Assefa F, Kim JA, Lim J, Nam SH, Shin HI, Park EK. The neuropeptide spexin promotes the osteoblast differentiation of MC3T3-E1 cells via the MEK/ERK pathway and bone regeneration in a mouse calvarial defect model. Tissue Eng Regen Med 2022; 19:189–202. 34951679 10.1007/s13770-021-00408-2 PMC8782952

[17] Chen Y, He M, Lei MML, Ko WKW, Lin C, Bian Z, Wong AOL. Mouse spexin: (III) differential regulation by glucose and insulin in glandular stomach and functional implication in feeding control. Front Endocrinol 2021; 12:e681648. 10.3389/fendo.2021.681648. PMC8138665 34025589

[18] Deng SP, Chen HP, Zhai Y, Jia LY, Liu JY, Wang M, Jiang DN, Wu TL, Zhu CH, Li GL. Molecular cloning, characterization and expression analysis of spexin in spotted scat (*Scatophagus argus*). Gen Comp Endocrinol 2018; 266:60–66. 29753927 10.1016/j.ygcen.2018.04.018

[19] Li S, Liu Q, Xiao L, Chen H, Li G, Zhang Y, Lin H. Molecular cloning and functional characterization of spexin in orange-spotted grouper (*Epinephelus coioides*). Comp Biochem Physiol B Biochem Mol Biol 2016; 196-197:85–91. 26944307 10.1016/j.cbpb.2016.02.009

[20] Respekta N, Maślanka A, Mlyczyńska E, Billert M, Szlaga A, Sambak P, Pawlicki P, Płachno B, Skrzypski M, Kotula-Balak M, Błasiak A, Rak A. Levels of spexin and its receptors GALR2 and GALR3 in the hypothalamus and ovary of letrozole-induced polycystic ovary syndrome in rats. Biochem Biophys Res Commun 2022; 627:207–213. 36055012 10.1016/j.bbrc.2022.08.059

[21] Pałasz A, Suszka-Świtek A, Kaśkosz A, Plewka D, Bogus K, Filipczyk Ł, Błaszczyk I, Bacopoulou F, Worthington JJ, Piwowarczyk-Nowak A, Tyszkiewicz-Nwafor M, Wiaderkiewicz R. Spexin-expressing neurons in the magnocellular nuclei of the human hypothalamus. J Chem Neuroanat 2021; 111:101883. 33161073 10.1016/j.jchemneu.2020.101883

[22] Liu Y, Li S, Qi X, Zhou W, Liu X, Lin H, Zhang Y, Cheng CH. A novel neuropeptide in suppressing luteinizing hormone release in goldfish *Carassius auratus*. Mol Cell Endocrinol 2013; 374:65–72. 23623870 10.1016/j.mce.2013.04.008

[23] Cohen Y, Hausken K, Bonfil Y, Gutnick M, Levavi-Sivan B. Spexin and a novel cichlid-specific spexin paralog both inhibit FSH and LH through a specific galanin receptor (Galr2b) in tilapia. Front Endocrinol 2020; 11:e71. 10.3389/fendo.2020.00071 PMC7044129 32153508

[24] Guler A, Demir İ. Decreased levels of spexin are associated with hormonal and metabolic disturbance in subjects with polycystic ovary syndrome. J Obstet Gynaecol 2021; 41:408–413. 32293212 10.1080/01443615.2020.1737660

[25] March WA, Moore VM, Willson KJ, Phillips DI, Norman RJ, Davies MJ. The prevalence of polycystic ovary syndrome in a community sample assessed under contrasting diagnostic criteria. Hum Reprod 2010; 25:544–551. 19910321 10.1093/humrep/dep399

[26] Hoeger KM, Dokras A, Piltonen T. Update on PCOS: consequences, challenges, and guiding treatment. J Clin Endocrinol Metab 2021; 106:e1071–e1083. 33211867 10.1210/clinem/dgaa839

[27] Zeng X, Xie YJ, Liu YT, Long SL, Mo ZC. Polycystic ovarian syndrome: correlation between hyperandrogenism, insulin resistance and obesity. Clin Chim Acta 2020; 502:214–221. 31733195 10.1016/j.cca.2019.11.003

[28] Geng X, Zhao J, Huang J, Li S, Chu W, Wang WS, Chen ZJ, Du Y. Lnc-MAP3K13-7:1 inhibits ovarian GC proliferation in PCOS via DNMT1 downregulation-mediated CDKN1A promoter hypomethylation. Mol Ther 2021; 29:1279–1293. 33212300 10.1016/j.ymthe.2020.11.018 PMC7934583

[29] Baillargeon JP, Carpentier A. Role of insulin in the hyperandrogenemia of lean women with polycystic ovary syndrome and normal insulin sensitivity. Fertil Steril 2007; 88:886–893. 17559844 10.1016/j.fertnstert.2006.12.055 PMC3846535

[30] Beyazit F, Hiz MM, Turkon H, Unsal MA. Serum spexin, adiponectin and leptin levels in polycystic ovarian syndrome in association with FTO gene polymorphism. Ginekol Pol 2021; 92:682–688. 33914321 10.5603/GP.a2020.0176

[31] Franik G, Sadlocha M, Madej P, Owczarek A, Skrzypulec-Plinta V, Plinta R, Chudek J, Olszanecka-Glinianowicz M. Circulating omentin-1 levels and inflammation in polycystic ovary syndrome. Ginekol Pol 2020; 91:308–312. 32627151 10.5603/GP.2020.0057

[32] Bannigida DM, Nayak SB, R V. Serum visfatin and adiponectin - markers in women with polycystic ovarian syndrome. Arch Physiol Biochem 2020; 126:283–286. 30318936 10.1080/13813455.2018.1518987

[33] Namavar Jahromi B, Dabaghmanesh MH, Parsanezhad ME, Fatehpoor F. Association of leptin and insulin resistance in PCOS: a case-controlled study. Int J Reprod Biomed 2017; 15:423–428. 29177243 PMC5601933

[34] Bongrani A, Mellouk N, Ramé C, Cornuau M, Guerif F, Froment P, Dupont J. Vaspin, a novel adipokine in woman granulosa cells physiology and PCOS pathogenesis? J Endocrinol 2021; 249:57–70. 33608490 10.1530/JOE-20-0550

[35] Guerif F, Saussereau MH, Barthelemy C, Couet ML, Gervereau O, Lansac J, Royere D, Royere D. Efficacy of IVF using frozen donor semen in cases of previously failed DI cycles compared with tubal infertility: a cohort study. Reprod Biomed Online 2004; 9:404–408. 15511340 10.1016/s1472-6483(10)61275-8

[36] Nishi Y, Yanase T, Mu Y, Oba K, Ichino I, Saito M, Nomura M, Mukasa C, Okabe T, Goto K, Takayanagi R, Kashimura Y, et al. Establishment and characterization of a steroidogenic human granulosa-like tumor cell line, KGN, that expresses functional follicle-stimulating hormone receptor. Endocrinology 2001; 142:437–445. 11145608 10.1210/endo.142.1.7862

[37] Yu M, Wang M, Han S, Han L, Kan Y, Zhao J, Yu X, Yan J, Jin Y, Zhang Z, Shang W, Fang P. Spexin ameliorates skeletal muscle insulin resistance through activation of GAL2 receptor. Eur J Pharmacol 2022; 917:174731. 10.1016/j.ejphar.2021.174731. 34973950

[38] Kurowska P, Mlyczyńska E, Dawid M, Opydo-Chanek M, Dupont J, Rak A. In vitro effects of vaspin on porcine granulosa cell proliferation, cell cycle progression, and apoptosis by activation of GRP78 receptor and several kinase signaling pathways including MAP3/1, AKT, and STAT3. Int J Mol Sci 2019; 20:e5816. 10.3390/ijms20225816 PMC6888539 31752432

[39] Cloix L, Reverchon M, Cornuau M, Froment P, Ramé C, Costa C, Froment G, Lecomte P, Chen W, Royère D, Guerif F, Dupont J. Expression and regulation of INTELECTIN1 in human granulosa-lutein cells: role in IGF-1-induced steroidogenesis through NAMPT. Biol Reprod 2014; 91:e50. 10.1095/biolreprod.114.120410 24943040

[40] Kotula-Balak M, Gorowska-Wojtowicz E, Milon A, Pawlicki P, Tworzydlo W, Płachno BJ, Krakowska I, Hejmej A, Wolski JK, Bilinska B. Towards understanding leydigioma: do G protein-coupled estrogen receptor and peroxisome proliferator-activated receptor regulate lipid metabolism and steroidogenesis in Leydig cell tumors? Protoplasma 2020; 257:1149–1163. 32180008 10.1007/s00709-020-01488-y PMC7329793

[41] Greenbaum D, Colangelo C, Williams K, Gerstein M. Comparing protein abundance and mRNA expression levels on a genomic scale. Genome Biol 2003; 4:e117. 10.1186/gb-2003-4-9-117. PMC193646 12952525

[42] Kim A, Park T. Diet-induced obesity regulates the galanin-mediated signaling cascade in the adipose tissue of mice. Mol Nutr Food Res 2010; 54:1361–1370. 20183829 10.1002/mnfr.200900317

[43] Faure-Virelizier C, Croix D, Bouret S, Prévot V, Reig S, Beauvillain JC, Mitchell V. Effects of estrous cyclicity on the expression of the galanin receptor gal-R1 in the rat preoptic area: a comparison with the male. Endocrinology 1998; 139:4127–4139. 9751492 10.1210/endo.139.10.6271

[44] Tran A, Loganathan N, McIlwraith EK, Belsham DD. Palmitate and nitric oxide regulate the expression of spexin and galanin receptors 2 and 3 in hypothalamic neurons. Neuroscience 2020; 447:41–52. 31730796 10.1016/j.neuroscience.2019.10.028

[45] Baranowska B, Radzikowska M, Wasilewska-Dziubińska E, Kapliński A, Roguski K, Płonowski A, Neuropeptide Y. Leptin, galanin and insulin in women with polycystic ovary syndrome. Gynecol Endocrinol 1999; 13:344–351. 10599552 10.3109/09513599909167578

[46] Bouret S, Prevot V, Croix D, Howard A, Habert-Ortoli E, Jegou S, Vaudry H, Beauvillain JC, Mitchell V. Expression of GalR1 and GalR2 galanin receptor messenger ribonucleic acid in proopiomelanocortin neurons of the rat arcuate nucleus: effect of testosterone. Endocrinology 2000; 141:1780–1794. 10803589 10.1210/endo.141.5.7469

[47] Gajewska A, Zielinska-Gorska M, Wasilewska-Dziubinska E, Baran M, Kotarba G, Gorski K. Pituitary galaninergic system activity in female rats: the regulatory role of gonadal steroids. J Physiol Pharmacol 2016; 67:423–429. 27512003

[48] Li X, Zhu Q, Wang W, Qi J, He Y, Wang Y, Lu Y, Wu H, Ding Y, Sun Y. Elevated chemerin induces insulin resistance in human granulosa-lutein cells from polycystic ovary syndrome patients. FASEB J 2019; 33:11303–11313. 31311314 10.1096/fj.201802829R

[49] Hussein MR, Bedaiwy MA, Falcone T. Analysis of apoptotic cell death, Bcl-2, and p53 protein expression in freshly fixed and cryopreserved ovarian tissue after exposure to warm ischemia. Fertil Steril 2006; 85:1082–1092. 16616078 10.1016/j.fertnstert.2005.10.020

[50] Reverchon M, Cornuau M, Ramé C, Guerif F, Royère D, Dupont J. Chemerin inhibits IGF-1-induced progesterone and estradiol secretion in human granulosa cells. Hum Reprod 2012; 27:1790–1800. 22447628 10.1093/humrep/des089

[51] Yoshimura Y . Insulin-like growth factors and ovarian physiology. J Obstet Gynaecol Res 1998; 24:305–323. 9879150 10.1111/j.1447-0756.1998.tb00103.x

[52] Kurowska P, Gaździk K, Jasińska A, Mlyczyńska E, Wachowska D, Rak A. Resistin as a new player in the regulation of porcine corpus luteum luteolysis: in vitro effect on proliferation/viability, apoptosis and autophagy. J Physiol Pharmacol 2023; 74:21–30. 10.26402/jpp.2023.1.03 37245230

[53] Kruszewska J, Laudy-Wiaderny H, Kunicki M. Review of novel potential insulin resistance biomarkers in PCOS patients-the debate is still open. Int J Environ Res Public Health 2022; 19:2099. 35206286 10.3390/ijerph19042099 PMC8871992

[54] Giustina A, Gastaldi C, Bugari G, Chiesa L, Loda G, Tironi C, Negro-Vilar A. Role of galanin in the regulation of somatotrope and gonadotrope function in young ovulatory women. Metabolism 1995; 44:1028–1032. 7543651 10.1016/0026-0495(95)90100-0

[55] Reverchon M, Cornuau M, Cloix L, Ramé C, Guerif F, Royère D, Dupont J. Visfatin is expressed in human granulosa cells: regulation by metformin through AMPK/SIRT1 pathways and its role in steroidogenesis. Mol Hum Reprod 2013; 19:313–326. 23315983 10.1093/molehr/gat002

[56] Mason H, Franks S. Local control of ovarian steroidogenesis. Baillieres Clin Obstet Gynaecol 1997; 11:261–279. 9536211 10.1016/s0950-3552(97)80037-5

[57] Delitala AP, Capobianco G, Delitala G, Cherchi PL, Dessole S. Polycystic ovary syndrome, adipose tissue and metabolic syndrome. Arch Gynecol Obstet 2017; 296:405–419. 28643028 10.1007/s00404-017-4429-2

[58] Barbe A, Bongrani A, Mellouk N, Estienne A, Kurowska P, Grandhaye J, Elfassy Y, Levy R, Rak A, Froment P, Dupont J. Mechanisms of adiponectin action in fertility: an overview from gametogenesis to gestation in humans and animal models in normal and pathological conditions. Int J Mol Sci 2019; 20:1526. 30934676 10.3390/ijms20071526 PMC6479753

[59] Chabrolle C, Tosca L, Dupont J. Regulation of adiponectin and its receptors in rat ovary by human chorionic gonadotrophin treatment and potential involvement of adiponectin in granulosa cell steroidogenesis. Reproduction 2007; 133:719–731. 17504916 10.1530/REP-06-0244

[60] Hierlihy AM, Cooke GM, Curran IH, Mehta R, Karamanos L, Price CA. Effects of ciprofibrate on testicular and adrenal steroidogenic enzymes in the rat. Reprod Toxicol 2006; 22:37–43. 16337773 10.1016/j.reprotox.2005.11.001

[61] Wang M, Zhu Z, Kan Y, Yu M, Guo W, Ju M, Wang J, Yi S, Han S, Shang W, Zhang Z, Zhang L, et al. Treatment with spexin mitigates diet-induced hepatic steatosis in vivo and in vitro through activation of galanin receptor 2. Mol Cell Endocrinol 2022; 552:111688. 35654225 10.1016/j.mce.2022.111688

[62] Carpentier JL . Insulin receptor internalization: molecular mechanisms and physiopathological implications. Diabetologia 1994; 37:S117–S124. 7821727 10.1007/BF00400835

[63] Hegyi K, Fülöp K, Kovács K, Tóth S, Falus A. Leptin-induced signal transduction pathways. Cell Biol Int 2004; 28:159–169. 14984741 10.1016/j.cellbi.2003.12.003

[64] Kurowska P, Mlyczyńska E, Dawid M, Dupont J, Rak A. Role of vaspin in porcine ovary: effect on signaling pathways and steroid synthesis via GRP78 receptor and protein kinase A†. Biol Reprod 2020; 102:1290–1305. 32149334 10.1093/biolre/ioaa027 PMC7703729

[65] Kurowska P, Sroka M, Dawid M, Mlyczyńska E, Respekta N, Jurek M, Klimczyk D, Grzesiak M, Dupont J, Rak A. Expression and role of resistin on steroid secretion in the porcine corpus luteum. Reproduction 2021; 162:237–248. 34314376 10.1530/REP-21-0236

[66] Roche J, Ramé C, Reverchon M, Mellouk N, Cornuau M, Guerif F, Froment P, Dupont J. Apelin (APLN) and apelin receptor (APLNR) in human ovary: expression, signaling, and regulation of steroidogenesis in primary human luteinized granulosa cells. Biol Reprod 2016; 95:e104. 10.1095/biolreprod.116.141754. 27683264

[67] Maillard V, Uzbekova S, Guignot F, Perreau C, Ramé C, Coyral-Castel S, Dupont J. Effect of adiponectin on bovine granulosa cell steroidogenesis, oocyte maturation and embryo development. Reprod Biol Endocrinol 2010; 8:23. 20219117 10.1186/1477-7827-8-23 PMC2845137

[68] Cheng L, Shi H, Jin Y, Li X, Pan J, Lai Y, Lin Y, Jin Y, Roy G, Zhao A, Li F. Adiponectin deficiency leads to female subfertility and ovarian dysfunctions in mice. Endocrinology 2016; 157:4875–4887. 27700136 10.1210/en.2015-2080

