# Supplementary material for: Spexin role in human granulosa cells physiology and PCOS: expression and negative impact on steroidogenesis and proliferation
Source: Biol Reprod. 2023 Sep 2;109(5):705–19. doi: 10.1093/biolre/ioad108 (PMC10651070; doi:10.1093/biolre/ioad108)
Supplement: Supplementary_Table_1_ioad108 [file supplementary_table_1_ioad108.docx]

Supplementary Table 1: Characterization of patients.

| **Parameters** | **Normal weight** | **Obese** | **Normal weight PCOS** | **Obese**  **PCOS** |
| --- | --- | --- | --- | --- |
| BMI | 21.1 ± 0.42 | 32.2 ± 0.49 | 20.72 ± 0.43 | 33.2 ± 0.58 |
| Antral follicle count | 17.4 ± 1.41 | 9.4 ± 1.17 | 45.09 ± 4.26 | 31.4 ± 1.06 |
| Testosterone (μg/L) | 0.11 ± 0.02 | 0.15 ± 0.02 | 0.805 ± 0.077 | 0.78 ± 0.08 |
| FSH (UI/L) | 7.02 ± 0.92 | 6.79 ± 0.92 | 5.98 ± 0.34 | 4.98 ± 0.47 |
| LH (UI/L) | 4.06 ± 0.25 | 3.69 ± 0.43 | 8.88 ± 1.25 | 6.69 ± 0.71 |
| AMH (ng/mL) | 3.34 ± 0.46 | 4.1 ± 0.49 | 20.44 ± 4.71 | 10.25 ± 1.60 |
