# Supplementary material for: Spexin role in human granulosa cells physiology and PCOS: expression and negative impact on steroidogenesis and proliferation
Source: Biol Reprod. 2023 Sep 2;109(5):705–19. doi: 10.1093/biolre/ioad108 (PMC10651070; doi:10.1093/biolre/ioad108)
Supplement: Supplementary_Table_2_ioad108 [file supplementary_table_2_ioad108.docx]

Supplementary Table 2: Reagents used.

| **Regent** | **Product no** | **Vendor** |
| --- | --- | --- |
| ***Cell culture and in vitro experiments*** | | |
| Percoll | 17544501 | Cytiva, MA, USA |
| McCoy | M8403USA | Sigma-Aldrich, MO,USA |
| DMEM | CM10MEG8-01 | Eurobio Scientific, France |
| SPX | 023-81 | Phoenix Pharmaceuticals, Germany |
| IGF1 | I3769 | Merck, Germany |
| FSH | F4021 | Merck, Germany |
| PD98059 | 1213 | Tocris, GB |
| LY294002 | 9901 | Cell Signalling Technology, MA, USA |
| AG490 | T3434 | Sigma-Aldrich, MO, USA |
| KT570 | K3761 | Sigma-Aldrich, MO, USA |
| GALR2 siRNA | 4392420 | ThermoFisher Scientific, MA, USA |
| GALR3 siRNA | 4392420 | ThermoFisher Scientific, MA, USA |
| Negative control siRNA | 4390843 | ThermoFisher Scientific, MA, USA |
| RNAiMAX | 13778030 | ThermoFisher Scientific, MA, USA |
| ***Reverse transcription and qRT-PCR*** | | |
| Reverse transcription kit | M1705 | Promega, WI, USA |
| SYBR Green | 1708887 | BioRad, CA, USA |
| ***Western blot*** | | |
| WesternBright Quantum HRP | K-12043 D20 | Advansta Inc., CA, USA |
| ***ELISA*** | | |
| SPX | EK-023-81 | Phoenix Pharmaceuticals, Germany |
| P4 | EIA-1561 | DRG Instruments GmbH, Germany |
| E2 | EIA-2693 | DRG Instruments GmbH, Germany |
| ***Immunohistochemistry*** | | |
| Ovary section | HuFPT076 | Biomax, MD, USA |
| IgG rabbit | I8140 | Sigma-Aldrich, MO, USA |
| IgG goat | I9140 | Sigma-Aldrich, MO, USA |
| ***Cells proliferation*** | | |
| alamarBlue | DAL1100 | Invitrogen, Carlsbad, CA, USA |
