# Supplementary material for: Spexin role in human granulosa cells physiology and PCOS: expression and negative impact on steroidogenesis and proliferation
Source: Biol Reprod. 2023 Sep 2;109(5):705–19. doi: 10.1093/biolre/ioad108 (PMC10651070; doi:10.1093/biolre/ioad108)
Supplement: Supplementary_Table_3_ioad108 [file supplementary_table_3_ioad108.docx]

Supplementary Table 3: Antibodies used for western blot and immunohistochemistry.

| **Antibody** | **Host species** | **Dilution** | **RRID** | **Vendor** |
| --- | --- | --- | --- | --- |
| GALR2 | Rabbit | 1:200 | - | Abcam, GB product no. ab203072 |
| GALR3 | Goat | 1:200 | AB_880162 | Abcam, GB product no. ab48008 |
| pMAP3/1 | Rabbit | 1:300 | AB_331646 | Cell Signaling Technology, USA product no. #9101S |
| MAP3/1 | Rabbit | 1:300 | AB_330744 | Cell Signaling Technology, USA product no. #9102S |
| pAKT | Rabbit | 1:300 | AB_329825 | Cell Signaling Technology, USA product no. #9271S |
| AKT | Rabbit | 1:300 | AB_329827 | Cell Signaling Technology, USA product no. #9272S |
| pSTAT3 | Rabbit | 1:300 | AB_331586 | Cell Signaling Technology, USA product no. #9131S |
| STAT3 | Rabbit | 1:300 | AB_331269 | Cell Signaling Technology, USA product no. #4904S |
| pPKA | Rabbit | 1:300 | AB_305132 | Abcam, GB product no. ab5815 |
| PKA | Rabbit | 1:300 | - | Abcam, GB product no. ab187515 |
| PCNA | Mouse | 1:300 | AB_2533016 | ThermoFisher Scientific, USA, product no. 13-3900 |
| STAR | Rabbit | 1:500 | - | Abcam, GB product no. ab233427 |
| CYP11A1 | Goat | 1:200 | AB_2088369 | Santa Cruz Biotechnology, USA product no. sc-18040 |
| CYP17A1 | Goat | 1:1000 | AB_638927 | Santa Cruz Biotechnology, USA product no. sc-46084 |
| HSD3B | Mouse | 1:1000 | AB_2631971 | Abcam, GB product no. ab75710 |
| HSD17B | Goat | 1:1000 | AB_2119665 | Santa Cruz Biotechnology, USA product no. sc-26963 |
| CYP19A1 | Rabbit | 1:200 | AB_2088676 | ThermoFisher Scientific, USA product no. PA1-21398 |
| ACTB | Mouse | 1:5000 | AB_476743 | Sigma-Aldrich, USA product no. A5316 |
| TUBA | Rabbit | 1:1000 | AB_2210206 | Proteitech, GB, 11224-1-AP |
| ANTI-MOUSE | Horse | 1:1000 | AB_330924 | Cell Signaling Technology, USA product no. #7076 |
| ANTI-RABBIT | Goat | 1:1000 | AB_2099233 | Cell Signaling Technology, USA product no. #7074 |
| ANTI-GOAT | Mouse | 1:1000 | AB_628490 | Santa Cruz Biotechnology, USA product no. sc-2354 |
