# Supplementary material for: Spexin role in human granulosa cells physiology and PCOS: expression and negative impact on steroidogenesis and proliferation
Source: Biol Reprod. 2023 Sep 2;109(5):705–19. doi: 10.1093/biolre/ioad108 (PMC10651070; doi:10.1093/biolre/ioad108)
Supplement: Supplementary_Table_4_ioad108 [file supplementary_table_4_ioad108.docx]

Supplementary Table 4: Statistically relevant *P* values between comparisons presented in the manuscript

Gene expression of SPX and GALR2/3 in granulosa cells and levels of SPX in FF

| **Normal weight** | **Obese** | **Normal weight PCOS** | **Obese**  **PCOS** | **KGN** |
| --- | --- | --- | --- | --- |
| ***SPX mRNA level*** | | | | |
| *-* | *P<0.0001* | *P<0.0001* | *P<0.0001* | *P=0.045* |
| ***GALR2 mRNA level*** | | | | |
| *-* | *ns* | *ns* | *ns* | *P<0.0001* |
| ***GALR3 mRNA level*** | | | | |
| *-* | *P<0.0001* | *P=0.001* | *P<0.0001* | *ns* |
| ***SPX FF level*** | | | | |
| *-* | *P<0.0001* | *P<0.0001* | *P<0.0001* | *Non investigated* |

Effect of SPX on PCNA mRNA levels

|  | **C** | **SPX**  **(0.1 nM)** | **SPX**  **(1 nM)** | **SPX**  **(10 nM)** | **SPX**  **(100 nM)** | **IGF1** | **IGF1**  **+ SPX (1nM)** | **FSH** | **FSH**  **+ SPX (1 nM)** |
| --- | --- | --- | --- | --- | --- | --- | --- | --- | --- |
| ***PCNA*** | | | | | | | | | |
| **KGN** | *-* | *ns* | *P<0.0001* | *P<0.0001* | *P<0.0001* | *-* | *P<0.0001* | *-* | *ns* |
| **Normal weight** | *-* | *ns* | *ns* | *P<0.0001* | *P<0.0001* | *-* | *P<0.0001* | *-* | *P<0.0001* |
| **Obese** | *-* | *ns* | *0.0010* | *P<0.0001* | *P<0.0001* | *-* | *P<0.0001* | *-* | *ns* |
| **Normal weight PCOS** | *-* | *ns* | *P<0.0001* | *P<0.0001* | *P<0.0001* | *-* | *P<0.0001* | *-* | *ns* |
| **Obese**  **PCOS** | *-* | *ns* | *ns* | *P<0.0001* | *P<0.0001* | *-* | *ns* | *-* | *ns* |

Effect of SPX on P4 and E2 secretion by granulosa cells

|  | **C** | **SPX**  **(0.1 nM)** | **SPX**  **(1 nM)** | **SPX**  **(10 nM)** | **SPX**  **(100 nM)** | **IGF1** | **IGF1**  **+ SPX (1nM)** | **FSH** | **FSH**  **+ SPX (1 nM)** |
| --- | --- | --- | --- | --- | --- | --- | --- | --- | --- |
| ***P4*** | | | | | | | | | |
| **KGN** | *-* | *ns* | *ns* | *ns* | *ns* | *-* | *ns* | *-* | *ns* |
| **Normal weight** | *-* | *ns* | *ns* | *ns* | *P<0.0001* | *-* | *ns* | *-* | *ns* |
| **Obese** | *-* | *ns* | *ns* | *P=0.0023* | *P=0.0008* | *-* | *ns* | *-* | *ns* |
| **Normal weight PCOS** | *-* | *ns* | *ns* | *ns* | *P=0.05* | *-* | *ns* | *-* | *ns* |
| **Obese**  **PCOS** | *-* | *ns* | *ns* | *P=0.0298* | *P=0.0109* | *-* | *ns* | *-* | *ns* |
| ***E2*** | | | | | | | | | |
| **KGN** | *-* | *ns* | *P=0.0025* | *P=0.0017* | *ns* | *-* | *ns* | *-* | *ns* |
| **Normal weight** | *-* | *ns* | *ns* | *ns* | *P<0.0001* | *-* | *ns* | *-* | *ns* |
| **Obese** | *-* | *ns* | *ns* | *P=0.0022* | *P=0.0002* | *-* | *ns* | *-* | *ns* |
| **Normal weight PCOS** | *-* | *ns* | *ns* | *P=0.0230* | *P=0.0068* | *-* | *ns* | *-* | *ns* |
| **Obese**  **PCOS** | *-* | *ns* | *ns* | *P=0.0164* | *P=0.0027* | *-* | *ns* | *-* | *ns* |

Effect of SPX on STAR protein and steroidogenic enzymes expression in KGN cells

|  | **C** | **SPX**  **(0.1 nM)** | **SPX**  **(1 nM)** | **SPX**  **(10 nM)** | **SPX**  **(100 nM)** | **IGF1** | **IGF1**  **+ SPX (1nM)** | **FSH** | **FSH**  **+ SPX (1 nM)** |
| --- | --- | --- | --- | --- | --- | --- | --- | --- | --- |
| ***StAR*** | *-* | *P=0.0089* | *ns* | *ns* | *ns* | *-* | *ns* | *-* | *ns* |
| ***CYP11A1*** | *-* | *ns* | *P<0.0001* | *P<0.0001* | *ns* | *-* | *P<0.0001* | *-* | *P<0.0001* |
| ***HSD3B*** | *-* | *ns* | *ns* | *ns* | *ns* | *-* | *ns* | *-* | *ns* |
| ***CYP17A1*** | *-* | *ns* | *P<0.0001* | *P<0.0001* | *ns* | *-* | *P<0.0001* | *-* | *P<0.0001* |
| ***HSD17B*** | *-* | *ns* | *ns* | *ns* | *ns* | *-* | *ns* | *-* | *ns* |
| ***CYP19A1*** | *-* | *ns* | *ns* | *ns* | *ns* | *-* | *ns* | *-* | *ns* |

Effect of SPX on HSD3B and CYP19A1 in human Gc

|  | **C** | **SPX**  **(0.1 nM)** | **SPX**  **(1 nM)** | **SPX**  **(10 nM)** | **SPX**  **(100 nM)** | **IGF1** | **IGF1**  **+ SPX (1nM)** | **FSH** | **FSH**  **+ SPX (1 nM)** |
| --- | --- | --- | --- | --- | --- | --- | --- | --- | --- |
| ***HSD3B*** | | | | | | | | | |
| **Normal weight** | *-* | *ns* | *P=0.003* | *P<0.0001* | *P<0.0001* | *-* | *P<0.0001* | *-* | *ns* |
| **Obese** | *-* | *ns* | *P<0.0001* | *P<0.0001* | *P<0.0001* | *-* | *ns* | *-* | *ns* |
| **Normal weight PCOS** | *-* | *ns* | *ns* | *P<0.0001* | *P<0.0001* | *-* | *ns* | *-* | *ns* |
| **Obese PCOS** | *-* | *ns* | *P=0.02* | *P<0.0001* | *P<0.0001* | *-* | *ns* | *-* | *ns* |
| ***CYP19A1*** | | | | | | | | | |
| ***Normal weight*** | *-* | *ns* | *ns* | *P<0.0001* | *P<0.0001* | *-* | *ns* | *-* | *ns* |
| ***Obese*** | *-* | *ns* | *P=0.009* | *P<0.0001* | *P<0.0001* | *-* | *ns* | *-* | *ns* |
| ***Normal weight PCOS*** | *-* | *ns* | *ns* | *P=0.0003* | *P<0.0001* | *-* | *ns* | *-* | *ns* |
| ***Obese PCOS*** | *-* | *ns* | *ns* | *P=0.0005* | *P<0.0001* | *-* | *ns* | *-* | *ns* |

Effectiveness of GALR2 and GALR3 silencing

| C | **siRNA (10 pM)** | **siRNA (15 pM)** | **siRNA (20 pM)** |
| --- | --- | --- | --- |
| ***GALR2 mRNA level*** | | | |
| *-* | *ns* | *P<0.0001* | *P<0.0001* |
| ***GALR3 mRNA level*** | | | |
| *-* | *P<0.0001* | *P<0.0001* | *P<0.0001* |
